# Comparison of sperm characteristics following single or double-layer density gradient centrifugation

**DOI:** 10.1186/s12610-026-00331-3

**Published:** 2026-11-10

**Authors:** Huidrom Yaiphaba Meitei, Dhakshanya Predheepan, Shubhashree Uppangala, Guruprasad Kalthur, Satish Kumar Adiga

**Affiliations:** 1 Department of Reproductive Science, Kasturba Medical College, Manipal Academy of Higher Education, Manipal, India; 2 Sree Mookambika Centre for Research and Innovation, Sree Mookambika Institutes, Padanilam, Kulasekharam, Tamil Nadu India

**Keywords:** DNA fragmentation, Male fertility, Semen quality, Sperm wash, Fragmentation de l’ADN, Fertilité masculine, Qualité du sperme, Lavage des spermatozoïdes

## Abstract

**Background:**

The present study aimed to understand whether single and double-layer density gradient systems provide differential sperm preparation outcomes. The prospective comparative study included semen samples from 25 normozoospermic and 25 non-normozoospermic men. Each sample was equally split and processed using single- and double-layer density gradient centrifugation, and the recovered spermatozoa were assessed for basic sperm characteristics, kinematics, and DNA integrity. The outcomes were compared between the two gradient systems in both groups.

**Results:**

Sperm motility, kinematics, mitochondrial membrane potential, and DNA integrity did not differ significantly in sperm fractions recovered using single- and double-layer density gradient systems in both normozoospermic and non-normozoospermic ejaculates. In normozoospermic ejaculates, total motility (mean difference, MD 2.1%, 95% CI -2.5 to 6.9; *p* = 0.36), mitochondrial membrane potential (MD -0.7, 95% CI -6.0 to 4.5, *p* = 0.77) and DNA integrity (MD -0.0, 95% -3.2 to 3.1; *p* = 0.98) was not statistically different between the gradient systems immediately post sperm selection. Similar findings were observed in non-normozoospermic ejaculates for total motility (MD 5.0%, 95% CI -4.9 to 14.9; *p* = 0.31), mitochondrial membrane potential (MD -3.3, 95% CI -18.1 to 11.5; *p* = 0.65) and DNA integrity (MD -3.0, 95% CI -7.6 to 1.6; *p* = 0.19). The findings remain unaltered after 6 h of incubation.

**Conclusion:**

Single-layer density gradient centrifugation effectively selects competent spermatozoa from both normozoospermic and non-normozoospermic samples, highlighting its potential as a technically simple and cost-effective approach for sperm processing.

## Résumé

**Contexte:**

La présente étude visait à déterminer si les systèmes de gradient de densité à une ou deux couches produisent des résultats différents en matière de préparation des spermatozoïdes. Cette étude comparative prospective a porté sur des échantillons de sperme provenant de 25 hommes normozoospermiques et de 25 hommes non normozoospermiques. Chaque échantillon a été divisé en deux fractions égales, puis traité par centrifugation sur gradient de densité à une ou deux couches. Les spermatozoïdes récupérés ont ensuite été évalués en termes de caractéristiques spermatiques de base, de paramètres cinématiques et d’intégrité de l’ADN. Les résultats obtenus avec les deux systèmes de gradient ont été comparés dans les deux groupes.

**Résultats:**

La mobilité, la cinématique, le potentiel de membrane mitochondriale et l’intégrité de l’ADN des spermatozoïdes ne différaient pas significativement entre les fractions spermatiques recueillies à l’aide de systèmes de gradient de densité monocouche ou bicouche, tant dans les éjaculats normozoospermiques que non normozoospermiques. Dans les éjaculats normozoospermiques, la mobilité totale (différence moyenne [DM] : 2,1% ; IC à 95% : −2,5 à 6,9 ; p = 0,36), le potentiel de membrane mitochondriale (DM : −0,7 ; IC à 95% : −6,0 à 4,5 ; p = 0,77) et l’intégrité de l’ADN (DM : −0,0 ; IC à 95% : −3,2 à 3,1 ; p = 0,98) ne présentaient pas de différence statistiquement significative entre les systèmes de gradient immédiatement après la sélection spermatique. Des résultats similaires ont été observés dans les éjaculats non normozoospermiques pour la mobilité totale (DM : 5,0% ; IC à 95% : −4,9 à 14,9 ; p = 0,31), le potentiel de membrane mitochondriale (DM : −3,3 ; IC à 95% : −18,1 à 11,5 ; p = 0,65) et l’intégrité de l’ADN (DM : −3,0 ; IC à 95% : −7,6 à 1,6 ; p = 0,19). Ces résultats demeuraient inchangés après 6 h d’incubation.

**Conclusion:**

La centrifugation sur gradient de densité monocouche permet de sélectionner efficacement les spermatozoïdes fonctionnellement compétents à partir d’échantillons normozoospermiques comme non normozoospermiques, ce qui souligne son potentiel en tant que méthode de préparation des spermatozoïdes techniquement simple et rentable.

## Background

Sperm preparation in assisted reproductive technology (ART) aims to enrich spermatozoa with optimal motility, morphology and DNA integrity while minimizing cellular damage. Alongside conventional methods such as density gradient centrifugation (DGC) and swim-up, advanced approaches including MACS, hyaluroate based selection techniques, microfluidics are increasingly explored. However, differences in processing complexity, accessibility and clinical applicability highlight the need to evaluate simple sperm processing strategies. DGC separates spermatozoa based on density and is widely used for sperm preparation techniques for assisted reproductive technology (ART) [1]. Multiple technical modifications have been explored to enhance sperm yield and functional competence, including variations in the number of gradient layers used [2] ranging from single [3], two [4], three [5, 6], four [7] and even 10-layer [8] protocols. While double-layer DGC (DL-DGC) is commonly applied in human ART, single-layer DGC (SL-DGC) has been extensively used in livestock [9–11] but remains inadequately evaluated for human ejaculates. SL-DGC offers potential advantages by reducing technical complexity and cost; however, its ability to recover functionally competent spermatozoa requires validation. Therefore, this study compared SL-DGC and DL-DGC with respect to sperm yield and functional competence in normozoospermic and non-normozoospermic ejaculates.

## Methods

### Study population

Fifty residual ejaculates from men undergoing fertility evaluation at the University Fertility Clinic were included following Institutional Ethics Committee approval (IEC 520/2019) and written informed consent. The study was conducted in accordance with the principles of the declaration of Helsinki and World Health Organization (WHO) guidelines. All the assessments were blinded with all evaluations being conducted by a single observer.

### Sperm preparation

Twenty-five normozoospermic and twenty-five non-normozoospermic ejaculates were analyzed, excluding samples with > 1 × 10⁶ round cells and grade III viscosity. The non-normozoospermic ejaculates comprise oligozoospermic (*n* = 11), asthenozoospermic (*n* = 3) and oligoasthenozoospermic (*n* = 11). Each ejaculate was divided into three aliquots: neat ejaculate (NE), SL-DGC (80%, ^V^Grad80, VITROMED, Germany), and DL-DGC (80/40%, ^V^Grad80 and ^V^Grad40, VITROMED, Germany). The gradients consisted silane coated silica particles. The densities of the 40% and 80% gradients were 1.050–1.061 g/cm^3^ and 1.100–1.111 g/cm^3^, respectively, whereas the osmolality ranged 310–340 mOsm/kg for both gradients. A 1:1 ratio of gradient medium to semen sample was used for sperm selection as per manufacturer’s instructions. Briefly, 0.5 mL aliquots of samples were carefully overlaid onto either the 40%/80% discontinuous gradient or the 80% gradient. The samples were centrifuged at 350 g for 20 min, washed twice at 300 g for 8 min in Earle’s Balanced Salt Solution supplemented with 0.1% Human Serum Albumin, and resuspended for immediate analysis. Additionally, the interval of 6 h post-processing of the ejaculate was considered to mimic the clinical utility in intrauterine insemination cycles where sperm oocyte interaction may not happen immediately, and sperm may have to wait in the fallopian tube until the arrival of the oocyte. Further, the longevity of the post-selected sperm cells was following 6 h of incubation at 37 °C and 5% CO_2_ as rapid method of Human sperm survival assay [12, 13]. Sperm concentration, motility, and morphology were assessed using WHO (2010) standards.

### Sperm kinematics assessment by CASA

Sperm kinematics were assessed as described earlier [14]. In brief, 8 µL of sperm suspension from each experimental group was taken on a pristine glass slide. Thirteen consecutive non-overlapping fields were captured, and sperm kinematics were assessed in 500 sperm cells at 37 °C using phase contrast microscope (UB200i) integrated with Computer Assisted Semen Analysis CASA software (Integrated Semen Analysis System; PROiSER, Spain) with a depth of 0.17 mm between the glass slide and the cover slip. Data on curvilinear velocity (VCL, µm/s), straight-line velocity (VSL, µm/s), average path velocity (VAP, µm/s), amplitude of lateral displacement (ALH, µm), linearity (LIN, %), straightness (STR, %), wobble (WOB, %), and beat-cross frequency (BCF, Hz) were captured. The percentage of hyperactivated sperm cells was considered with VCL, LIN and ALH.

### Mitochondrial membrane potential

Sperm mitochondrial potential was assessed as described earlier [14]. Processed fraction containing approximately 0.5 million spermatozoa was incubated with 2 µM JC-1 (5,5,6,6’-tetrachloro-1,1’,3,3’-tetraethylbenzimi-dazoylcarbocyanine iodide; Cat. No. T3168, Molecular Probes, Life 151 Technologies, USA) at 37 °C and 5% CO_2_. After 20 min, sperm cells were washed and evaluated under the fluorescence microscope (Imager A1, Carl Zeiss, Germany). Spermatozoa showing bright red to yellow fluorescence in the region of the midpiece were classified to have functional mitochondria. A minimum of 200 sperm cells were assessed from each group to determine the percentage of sperm exhibiting intact mitochondria.

### Sperm chromatin dispersion (SCD) test

Sperm chromatin dispersion assay was carried out on processed spermatozoa as described earlier [14]. A mixture of 0.1 million spermatozoa and 1% low-melting agarose kept at 37 °C was layered on slides pre-coated with 0.65% of normal melting agarose and allowed to solidify. The slides were immersed in denaturation solution (0.08 N HCl), lysis solution 1 (0.4 M Tris, 20mM DTT, 1% SDS, 50 mM EDTA), and lysis solution 2 (0.4 M Tris, 2 M NaCl). The slides were then air-dried after being neutralized in Tris buffer (0.4 M Tris) and serially dehydrated in alcohol. Sperm chromatin integrity was assessed under a fluorescent microscope (Imager-A1; Zeiss, Gottingen, Germany) after staining the cells with ethidium bromide (7 µg/mL). The large halo spermatozoa (without any DNA damage or normal) produce halos with a thickness equal to or greater than the length of the minor diameter of the core, whereas small halo spermatozoa produce halos with a thickness equal to or smaller than 1/3 the diameter of the minor diameter of the core. A total of 500 cells were assessed, and the percentage of spermatozoa with damaged DNA was calculated by counting spermatozoa with a small + no halo. The percentage of spermatozoa with severely damaged DNA was calculated by counting spermatozoa with no halo.

### Statistical analysis

Statistical analysis was performed by Jamovi (version 2.3) using repeated measures Analysis of Variance or Friedman’s test and Paired t-test or the Wilcoxon signed -rank test was used to compare the groups, as appropriate. Data are presented as mean ± SEM, and significance was set at *p* < 0.01.

## Results

The semen profile of the study subjects is shown in Table 1. The mean sperm recovery rate was not statistically different between the SL-DGC and DL-DGC in normozoospermic and non-normozoospermic cohorts. Similarly, the total and progressive motility at the 0th and 6th hour post-preparation were similar between the SL-DGC and DL-DGC. The sperm kinematics of the extracted fractions were similar between SL-DGC and DL-DGC at the 0th and 6th hour post preparation in both the cohorts. The percentage of motile spermatozoa and morphologically normal spermatozoa was significantly (*p* < 0.01) higher than that of neat ejaculates (NE) in both SL-DGC and DL-DGC, irrespective of ejaculate quality, while no difference was found between the two methods. The percentage spermatozoa with low mitochondrial membrane potential at 0th hour was significantly reduced in both SL-DGC and DL-DGC than those of the NE in both normozoospermic and non-normozoospermic cohorts (*p* < 0.001), but no difference was observed between the gradient systems. Importantly, the percentage of spermatozoa with DNA fragmentation was significantly (*p* < 0.001) reduced in both SL-DGC and DL-DGC than those of the NE in both normozoospermic and non-normozoospermic cohorts, but no difference was observed between the gradient systems. However, the percentage of spermatozoa with low mitochondrial membrane potential and high DNA fragmentation at 6th hour post-processing was not statistically different between the gradient systems in both normozoospermic and non-normozoospermic cohorts (Table 2).

**Table 1:** Age and semen profile of the study subjects

	Normozoospermic Mean ± SEM (*n* = 25)	Non-normozoospermic Mean ± SEM (*n* = 25)
Patient age (in year)	37.82 ± 1.01	37.17 ± 0.72
Semen volume (mL)	3.00 ± 0.28	2.89 ± 0.30
Sperm concentration (10^6^/mL)	51.35 ± 3.78	7.99 ± 1.70
Total sperm number (10^6^/ejaculate)	147.60 ± 15.38	21.87 ± 4.85
Total motility (%)	65.19 ± 1.62	40.66 ± 3.44
Progressive motility (%)	42.81 ± 2.45	22.43 ± 2.79
Normal forms (%)	5.31 ± 0.4	5.72 ± 0.5

**Table 2:** Function and quality of spermatozoa recovered by SL-DGC and DL-DGC in normozoospermic (*n* = 25) and non-normozoospermic (*n* = 25) samples

Normozoospermic									
Parameters	NE	Post extraction 0th hour				Post extraction 6th hour			
		SL-DGC	DL-DGC	MD 95% CI	*p*-value	SL-DGC	DL-DGC	MD 95% CI	*p*-value
Recovery rate (%)	-	34.5 ± 3.8 35.2 (6.7, 87.0)	40.9 ± 3.4 40.6 (11.5, 72.6)	6.4 (-3.8, 16.8)	0.21	-	-	-	-
Normal morphology (%)	5.3 ± 0.4 5.0 (4.0, 8.0)	9.6 ± 0.5^*^ 9.0 (8.0, 12.0)	10.4 ± 0.8^*^ 10.0 (6.0, 15.0)	0.7 (-1.1, 2.7)	0.41	-	-	-	-
Total motility (%)	65.1 ± 1.6 64.0 (42.0, 78.0)	73.6 ± 1.9^*^ 74.0 (51.0, 87.5)	75.4 ± 1.7^*^ 76.7 (59.4, 89.6)	2.1 (-2.5, 6.9)	0.36	58.1 ± 2.3 58.6 (36.0, 79.2)	59.5 ± 2.3 61.6 (34.6, 75.7)	2.4 (-3.5, 8.3)	0.42
Progressive motility (%)	42.8 ± 2.5 43.0 (17.0, 64.0)	49.0 ± 2.6 45.6 (25.0, 73.1)	50.4 ± 2.6^*^ 47.5 (21.5, 76.8)	1.0 (-6.0, 8.1)	0.77	34.1 ± 1.9 32.0 (10.0, 56.9)	34.0 ± 1.8 30.6 (18.8, 49.5)	0.3 (-4.2, 5.5)	0.88
Low mitochondrial membrane potential (%)	38.4 ± 2.2 36.8 (25.6, 50.7)	26.8 ± 1.9^**^ 25.8 (16.3, 41.8)	26.1 ± 1.7^**^ 25.0 (15.5, 38.5)	-0.7 (-6.0, 4.5)	0.77	41.9 ± 3.0 42.8 (19.8, 58.4)	39.4 ± 2.6 40.0 (20.0, 53.9)	-2.4 (-10.5, 5.6)	0.54
DNA fragmentation (%)	25.9 ± 0.8 26.1 (18.7, 34.5)	18.6 ± 1.1^**^ 18.9 (7.7, 34.3)	18.6 ± 1.6^**^ 18.6 (8.1, 33.9)	-0.0 (-3.2, 3.1)	0.98	35.0 ± 1.8 33.9 (17.8, 62.2)	30.5 ± 1.7 30.6 (17.3, 51.5)	-4.4 (-9.4, 0.5)	0.07
Curvilinear velocity (VCL; µm/s)	-	39.5 ± 3.7 37.5 (26.0, 60.0)	40.3 ± 3.5 40.0 (26.0, 60.0)	0.8 (-10.0, 11.6)	0.87	56.1 ± 5.4 53.0 (36.0, 80.0)	54.8 ± 4.1 54.5 (40.0, 77.0)	-1.3 (-15.6, 13.0)	0.85
Straight-line velocity (VSL; µm/s)	-	15.3 ± 1.0 15.0 (9.0, 20.0)	16.3 ± 1.1 16.5 (10.0, 23.0)	1.0 (-2.1, 4.1)	0.51	19.5 ± 1.4 19.5 (14.0, 26.0)	19.1 ± 0.5 19.5 (16.0, 21.0)	-0.4 (-3.3, 2.5)	0.78
Average path velocity (VAP; µm/s)	-	24.4 ± 1.6 23.5 (17.0, 31.0)	24.9 ± 1.4 25.0 (19.0, 32.0)	0.5 (-3.9, 4.9)	0.40	30.4 ± 2.0 30.5 (22.0, 42.0)	29.5 ± 1.3 30.0 (25.0, 37.0)	-0.9 (-5.9, 4.1)	0.35
Amplitude of lateral displacement (ALH; µm)	-	1.1 ± 0.2 1.0 (0.0, 2.0)	1.7 ± 0.3 1.0 (1.0, 2.0)	0.2 (-0.3, 0.7)	0.42	1.3 ± 0.2 1.0 (1.0, 3.0)	1.9 ± 0.2 2.0 (1.0, 3.0)	0.2 (-0.5, 0.9)	0.57
Linearity (LIN; %)	-	41.2 ± 3.1 41.5 (25.0, 53.0)	42.3 ± 2.9 40.0 (30.0, 57.0)	1.1 (-7.8, 10.0)	0.79	36.5 ± 2.8 34.0 (28.0, 56.0)	37.4 ± 2.5 36.0 (26.0, 49.0)	0.9 (-6.8, 8.6)	0.81
Straightness (STR; %)	-	64.2 ± 3.9 67.5 (44.0, 77.0)	66.0 ± 3.0 65.5 (53.0, 80.0)	1.8 (-8.5, 12.1)	0.71	64.8 ± 2.1 63.0 (58.0, 78.0)	65.2 ± 2.3 65.0 (54.0, 77.0)	0.4 (-6.1, 6.9)	0.89
Wobble (WOB; %)	-	63.9 ± 2.3 65.5 (51.0, 73.0)	63.5 ± 2.4 65.0 (49.0, 73.0)	-0.4 (-7.3, 6.5)	0.90	55.7 ± 2.5 56.5 (47.0, 71.0)	55.2 ± 2.2 54.0 (47.0, 65.0)	-0.5 (-7.5, 6.5)	0.88
Beat-cross frequency (BCF; Hz)	-	5.4 ± 0.6 6.0 (2.0, 8.0)	6.2 ± 0.5 6.5 (4.0, 9.0)	0.8 (-0.8, 2.4)	0.32	6.2 ± 0.4 6.0 (5.0, 8.0)	6.9 ± 0.2 7.0 (6.0, 8.0)	0.7 (-0.1, 1.5)	0.09
**Non-normozoospermic**									
**Parameters**	**NE**	**Post extraction 0th hour**				**Post extraction 6th hour**			
		**SL-DGC**	**DL-DGC**	**MD 95% CI**	**p-value**	**SL-DGC**	**DL-DGC**	**MD 95% CI**	**p-value**
Recovery rate (%)	-	37.6 ± 6.6 25.5 (3.0, 89.0)	45.2 ± 5.9 37.1 (6.7, 92.0)	7.6 (-10.0, 25.4)	0.38	-	-	-	-
Normal morphology (%)	5.7 ± 0.5 5.3 (4.0, 9.0)	8.6 ± 0.9^*^ 8.0 (5.0, 16.0)	9.0 ± 0.8^*^ 8.5 (5.0, 13.0)	0.4 (-1.9, 2.9)	0.70	-	-	-	-
Total motility (%)	40.6 ± 3.4 43.0 (2.0, 77.3)	52.3 ± 3.7^*^ 57.8 (8.0, 86.0)	57.3 ± 3.4^*^ 59.6 (10.0, 86.41)	5.0 (-4.9, 14.9)	0.31	37.6 ± 3.1 38.6 (0.0, 63.3)	42.6 ± 3.2 46.2 (0.0, 69.3)	5.0 (-3.7, 13.8)	0.25
Progressive motility (%)	22.4 ± 2.8 19.2 (0.0, 64.1)	24.5 ± 3.1 20.7 (0.0, 69.0)	26.5 ± 3.0 29.9 (0.0, 68.9)	1.9 (-6.5, 10.5)	0.64	16.1 ± 2.3 14.0 (0.0, 45.5)	18.7 ± 2.4 20.0 (0.0, 53.9)	2.5 (-3.9, 9.1)	0.43
Low mitochondrial membrane potential (%)	55.6 ± 4.6 51.2 (29.8, 88.0)	45.9 ± 4.9^**^ 39.7 (19.5, 79.6)	42.6 ± 5.4^**^ 36.5 (19.1, 77.4)	-3.3 (-18.1, 11.5)	0.65	57.7 ± 3.9 59.2 (33.9, 86.0)	49.6 ± 3.8 47.0 (28.5, 84.1)	-8.0 (-19.1, 2.9)	0.14
DNA fragmentation (%)	33.1 ± 1.8 31.5 (19.9, 56.1)	25.3 ± 1.8^**^ 25.7 (9.9, 43.6)	22.3 ± 1.5^**^ 20.9 (10.3, 36.2)	-3.0 (-7.6, 1.6)	0.19	40.4 ± 2.3 39.5 (19.7, 63.7)	36.2 ± 1.7 32.4 (23.9, 55.8)	-4.2 (-10.0, 1.6)	0.15
Curvilinear velocity (VCL; µm/s)	-	35.0 ± 3.7 33.0 (13.0, 58.0)	33.5 ± 3.5 33.0 (18.0, 49.0)	-1.4 (-13.0, 10.1)	0.79	37.0 ± 3.6 36.0 (23.0, 62.0)	36.7 ± 3.1 37.0 (24.0, 55.0)	-0.2 (-10.2, 9.6)	0.95
Straight-line velocity (VSL; µm/s)	-	15.2 ± 1.3 15.0 (8.0, 25.0)	16.9 ± 1.7 16.0 (11.0, 31.0)	1.6 (-2.8, 6.1)	0.45	14.8 ± 1.1 14.0 (11.0, 21.0)	14.7 ± 0.6 15.0 (11.0, 17.0)	-0.0 (-2.5, 2.4)	0.94
Average path velocity (VAP; µm/s)	-	22.1 ± 2.0 22.0 (10.0, 32.0)	21.6 ± 1.5 22.0 (15.0, 29.0)	-0.5 (-5.7, 4.6)	0.82	22.0 ± 1.5 22.0 (17.0, 31.0)	22.4 ± 1.2 23.0 (17.0, 29.0)	0.3 (-3.5, 4.2)	0.84
Amplitude of lateral displacement (ALH; µm)	-	1.0 ± 0.2 1.0 (0.0, 2.0)	1.1 ± 0.2 1.0 (0.0, 2.0)	0.18 (-0.3, 0.7)	0.49	1.4 ± 0.2 1.0 (1.0, 3.0)	1.2 ± 0.2 1.0 (1.0, 3.0)	-0.1 (-0.7, 0.4)	0.53
Linearity (LIN; %)	-	48.3 ± 4.3 49.0 (33.0, 78.0)	48.0 ± 3.3 46.0 (32.0, 65.0)	-0.3 (-11.6, 10.9)	0.94	41.7 ± 1.7 41.0 (33.0, 54.0)	42.3 ± 2.4 46.0 (28.0, 51.0)	0.63 (-5.4, 6.6)	0.82
Straightness (STR; %)	-	70.6 ± 2.5 72.0 (58.0, 82.0)	71.0 ± 1.9 72.0 (56.0, 79.0)	0.4 (-6.1, 7.0)	0.88	68.0 ± 1.1 67.0 (61.0, 76.0)	66.4 ± 2.1 69.0 (53.0, 75.0)	-1.5 (-6.4, 3.3)	0.52
Wobble (WOB; %)	-	67.3 ± 3.8 65.0 (53.0, 95.0)	66.0 ± 2.8 62.0 (57.0, 81.0)	-1.3 (-11.3, 8.5)	0.77	60.9 ± 1.8 60.0 (49.0, 71.0)	63.9 ± 1.9 62.0 (51.0, 72.0)	2.1 (-3.4, 7.7)	0.42
Beat-cross frequency (BCF; Hz)	-	5.0 ± 0.5 5.0 (2.0, 8.0)	4.9 ± 0.5 5.0 (2.0, 7.0)	-0.1 (-1.5, 1.2)	0.78	4.8 ± 0.5 4.0 (3.0, 7.0)	4.8 ± 0.4 5.0 (3.0, 7.0)	0.0 (-1.2, 1.2)	1.00

Values are in Mean ± SE and Median (Minimum, Maximum); NE-neat ejaculate; SL-DGC- Single layer density gradient centrifugation; DL-DGC- Double layer density gradient centrifugation; MD-Mean difference; CI-Confidence interval. p-value column represents the exact p-value obtained from the statistical comparison between SL-DGC and DL-DGC. *p < 0.01 vs. NE, **p < 0.001 vs. NE

## Discussion

The results presented here demonstrate that human spermatozoa selected using a single layer of silane-coated silica gradient exhibit sperm characteristics and quality was not statistically different to those obtained using the conventional double discontinuous gradient, in both normozoospermic and non-normozoospermic ejaculates. Clinically, these observations suggest that SL-DGC may provide a simpler approach for sperm preparation with favourable outcomes in sperm recovery rate, motility, morphology and functional longevity, irrespective of the ejaculate quality. Mitochondrial membrane potential, a key driver of sperm maturation, capacitation and acrosome reaction [15] was not statistically different between the two methods, indicating that SL-DGC is sufficient to enrich metabolically active spermatozoa. Historically, DL-DGC has been favoured due to its reported efficacy in reducing DNA fragmentation [2, 16] by isolating spermatozoa with compacted chromatin. Increase in DNA fragmentation at 6-hours may be attributed to prolonged in vitro incubation of processed spermatozoa, which has been shown to increase DNA fragmentation, possibly related to its length of epididymal transit [17, 18]. Previous study has reported 3–6-fold increase in the DNA fragmentation level in the processed fraction of sperm, at the end of six hours [16]. Interestingly, this study indicates that SL-DGC can achieve a similar reduction in DNA damage without necessitating the use of DL-DGC. We hypothesise that mechanical manipulation inherent to DL-DGC, including layering accuracy and migration of spermatozoa through the gradient interface, could potentially contribute to mechanical stress. SL-DGC may mitigate these risks by minimizing handling steps while maintaining selection efficacy. However, this hypothesis needs to be tested by additional experiments. Studies have reported that the sperm yield by DGC is influenced by semen viscosity and types of cells in the ejaculate [3]. The present study excluded the ejaculates with round cells > 1 million/mL and grade III viscosity Further, a limitation of this study is the heterogeneity of the non-normozoospermic group. Further, the baseline morphology was marginal in both cohorts, with normal forms close to the WHO lower reference limit. Given the narrow range of normal morphology values the broad range for detecting differences between processing methods may be limited. Owing to the limited sample size, subgroup analysis of specific semen abnormalities such as oligozoospermia, asthenozoospermia, teratozoospermia, and oligoasthenoteratozoospermia could not be performed. Also, the patient pathophysiological characteristics were either not available or not collected for the present study. These may have acted as a confounding factor, as the efficacy of the intervention may vary across different semen abnormalities and patient pathophysiological characteristics. Also, the study did not include the virus-infected samples, which may exhibit distinct density characteristics and partition differently during gradient centrifugation. Inclusion of such samples in future investigations, along with the impact on embryo development, quality and clinical outcomes, would further clarify the broader clinical applicability of SL-DGC.

## Conclusion

Collectively, these preliminary findings reveal that an additional gradient layer employed in DL-DGC does not confer an advantage in sperm selection. Consequently, SL-DGC appears to provide effective sperm selection while being technically simpler, and economically advantageous, reducing the gradient medium cost by approximately 50% per preparation, thereby supporting its potential use in andrology laboratories. Further studies are warranted to validate these findings and establish clinical value of SL-DGC in improving reproductive outcomes in ART.

## Data Availability

No datasets were generated or analysed during the current study.

## Abbreviations

DGC: Density gradient centrifugation
ART: Assisted Reproductive Technology
DL-DGC: Double-layer Density gradient centrifugation
SL-DGC: Single-layer Density gradient centrifugation
NE: Neat ejaculate
DNA: Deoxyribonucleic acid
JC-1: 5,5′,6,6′-tetrachloro-1,1′,3,3′-tetraethylbenzimidazolylcarbocyanine iodide
